# Cardiovascular Risk Factors and Their Relationship with Vascular Dysfunction in South African Children of African Ancestry

**DOI:** 10.3390/jcm10020354

**Published:** 2021-01-19

**Authors:** Edna N. Matjuda, Godwill A. Engwa, Samuel Nkeh Chungag Anye, Benedicta N. Nkeh-Chungag, Nandu Goswami

**Affiliations:** 1 Department of Human Biology, Faculty of Health Sciences, Walter Sisulu University PBX1, Mthatha 5117, South Africa; engoakoana@gmail.com; 2 Department of Biological and Environmental Sciences, Faculty of Natural Sciences, Walter Sisulu University PBX1, Mthatha 5117, South Africa; gengwa@wsu.ac.za; 3 MBCHB Programme, Faculty of Health Sciences, Walter Sisulu University PBX1, Mthatha 5117, South Africa; shmuelsam06@gmail.com; 4 Physiology Division, Otto Loewi Research Center for Vascular Biology, Immunology and Inflammation, Medical University of Graz, Neue Stiftingtalstrasse 6, 8036 Graz, Austria; nandu.goswami@medunigraz.at

**Keywords:** endothelial dysfunction, cardiovascular disease, arterial stiffness, obesity, hypertension, oxidative stress, microalbuminuria, vascular dysfunction

## Abstract

Vascular dysfunction is known to be an initiator of the development and progression of cardiovascular diseases (CVDs). However, there is paucity of information on the relationship of vascular dysfunction with cardiovascular risk factors in children of African ancestry. This study investigated the relationship between cardiovascular risk factors and vascular function in South African children of African ancestry. A cross-sectional study on 6–9-year-old children in randomly selected rural and urban schools of the Eastern Cape Province of South Africa was conducted. General anthropometric indices were measured, followed by blood pressure (BP) measurements. The pulse wave velocity (PWV) was measured using a Vicorder. Albumin to creatinine ratio (ACR), asymmetric dimethylarginine (ADMA), 8-hydroxy-2deoxyguanosine (8-OHdG) and thiobarbituric acid reactive substance (TBARS) were assayed in urine. Children from urban settings (10.8%) had a higher prevalence of overweight/obesity than their rural counterparts (8.5%) while the prevalence of elevated/high blood pressure was higher in rural (23.2%) than urban children (19.0%). Mean arterial blood pressure (MAP) and diastolic blood pressure (DBP) increased with increasing PWV (*p* < 0.05). Body mass index (BMI), diastolic blood pressure (DBP) and mean arterial blood pressure (MAP) positively associated (*p* < 0.05) with PWV. Creatinine, albumin and ACR significantly (*p* < 0.005) increased with increasing ADMA. ADMA associated positively (*p* < 0.05) with creatinine and 8-OHdG. In conclusion, vascular dysfunction was associated with obesity, high blood pressure, oxidative stress and microalbuminuria in South African children of African ancestry.

## 1. Introduction

Cardiovascular diseases (CVDs) are a worldwide problem because they lead to morbidity and mortality [1]. Endothelial dysfunction, which results from impaired synthesis of nitric oxide (NO) and constitutes vascular dysfunction, is known to be an early initiator of the progression of atherosclerosis and other CVDs [2,3]. One of the earliest signs of vascular damage or endothelial dysfunction is arterial stiffness, as stiffening of arteries is generally associated with changes in the endothelium of the arterial wall. It has been shown that impaired NO synthesis increases local arterial stiffness [4]. As such, pulse wave velocity (PWV), which is known to be a non-invasive measure for assessing arterial stiffness [5], can also be used as a surrogate method to assess endothelial dysfunction [6] as it has been shown to correlate with endothelial function in hypertensive patients and controls. [7]. Endothelial dysfunction [2] and arterial stiffness [3], which both lead to vascular dysfunction, are known to be related to several cardiovascular risk factors including smoking, obesity, hypertension, microalbuminuria, oxidative stress, etc.

Studies have shown that childhood obesity is associated with endothelial dysfunction. Obesity is known to promote low-grade inflammatory response [8] which induces the synthesis of arginase [9]. Arginase competes with endothelial nitric oxide synthase (eNOS) for its substrate, L-arginine, thereby inhibiting the production of NO [10]. Additionally, asymmetric dimethylarginine (ADMA) inhibits eNOS by competing with its substrate, L-arginine, thereby impairing the production of NO [11]. Thus, decreased availability of NO and increased ADMA leads to endothelial dysfunction [12]. More so, pro-inflammatory cytokines as a result of obesity can promote the generation of reactive oxygen species (ROS) [13]. ROS is reported to change the conformation of eNOS, making it less effective for NO synthesis with the consequent decrease in NO production [14]. In addition, ROS can react with NO converting them to more NO reactive species such as superoxide (O^2•^) and peroxynitrite (ONOO^•^), thereby reducing NO levels [14]. Thus, increased ROS, which is characteristic of oxidative stress, can promote vascular dysfunction.

Vascular dysfunction may also lead to hypertension. It has been suggested that endothelial dysfunction may cause some structural and functional changes in the microvascular wall with a predominant and deleterious constrictive tone leading to hypertension [15]. More so, release of endothelium-derived relaxing factors (EDRFs) in the endothelium is impaired by endothelial dysfunction resulting in vasoconstriction, which may lead to hypertension [16]. Microalbuminuria has been suggested to be an independent risk factor for vascular dysfunction [17]. Although it remains unclear, it has been suggested that glomerular leaking of albumin is a reflection of vascular damage, which denotes atherosclerosis [18].

In South Africa, there is evidence of an increase in cardiovascular risk factors including obesity and hypertension in children [19,20]. More so, endothelial dysfunction was shown to occur in children exposed to cardiovascular risk factors including a family history of hypertension [21]. However, there is limited information on the relationship of vascular function with cardiovascular risk factors in South Africa children of African ancestry who are presented with increased cardiovascular risk. Therefore, the present study was aimed to assess the relationship between cardiovascular risk factors and vascular function in South African children of African ancestry.

## 2. Methods

### 2.1. Study Population and Design

This was a stratified cross-sectional study that randomly recruited South African children of African ancestry aged 6–9 years from primary school in rural and urban areas of the Eastern Cape Province of South Africa. The children were recruited from primary schools in Libode, a rural area, and from Mthatha and East London, which are urban areas. The sample size was calculated using the formula: n = $\frac{z^{2}p\left(1-p\right)}{d^{2}},$ where; *n* = sample size, z^2^ = confidence interval, *p* = sample proportion, and *d* = desired precision. From the calculation, the sample size (*n*) = $\frac{1.96^{2}0.27\left(1-0.27\right)}{0.05^{2}}$ = 303.

### 2.2. Ethical Consideration

This study was conducted in accordance with the guidelines of the Helsinki Declaration (2008 reviewed version) as well as the local and national regulations of South Africa. Ethical approval was obtained from Walter Sisulu University Health Sciences Ethics Committee with approval number: 112/2018. After careful explanation of the purpose and aim of the study, written informed consent was obtained from the parents/legal guardians of the children before enrolment into the study. The study adhered to the standards of reporting and was in accordance with the National Data Protection Acts as the identity of the participants was kept confidential. There were no important changes in the methods after study commencement.

### 2.3. Inclusion/Exclusion Criteria

Children of African ancestry aged 6–9 years free from any cardiovascular and renal diseases were recruited for the study. Ill, physically challenged, individuals having any self-reported comorbidity or cardiovascular diseases and those who were not of African ancestry were excluded from the study.

### 2.4. Anthropometric Measurements

Anthropometric measurements were performed according to the International Standards for Anthropometric Assessments [22] on all the participants. Participants’ heights were measured using a wall-mounted Harpenden stadiometer and recorded to the nearest 0.1 centimetres (cm). The weight was measured using a wireless Tanita weight scale (BC1000, Tanita Corporation, Tokyo, Japan) connected to a computer. Personal details of the children, including sex, age and height, were entered into the computer and the body mass index (BMI) for each participant was determined. BMI was calculated from weight and height as weight/height^2^ (Kg/m^2^) and converted to percentiles for age, sex and height as underweight: <5th percentile, normal weight: ≥5th to <85th percentile, overweight: ≥85th to <95th percentile and obese: ≥95th percentile [23]. The waist circumference (WC), mid-upper arm circumference (MUAC), neck circumference (NC), ankle circumference (AC), calf circumference (CC) and thigh circumference (TC) were measured using an anthropometric tape in cm.

### 2.5. Blood Pressure Measurements

Blood pressure (BP) was measured using an automatic sphygmomanometer (Omron M500, HEM-7321-D, Omron Corporation, Kyoto, Japan). After resting for 5 min, a paediatric cuff attached to the sphygmomanometer was fitted to the bare right upper arm of the children and they sat upright on a chair with their right arm on the table. Three BP readings, which included the systolic blood pressure (SBP), diastolic blood pressure (DBP) and heart rate (HR), were taken at 2-min intervals. The average of the second and third BP readings was determined and converted to BP percentiles for sex, age and height and classified according to the American Academy of Paediatrics (AAP) 2017 guideline as normotensive: SBP and DBP < 90th percentile; Elevated BP: SBP and/or DBP > 90th < 95th percentile or high BP: SBP and/or DBP ≥ 95th percentile [24]. Mean arterial pressure (MAP) was calculated from the formula: MAP = (SBP + (2 × DBP))/3.

### 2.6. Vascular Function Assessment

Vascular function was assessed using a Vicorder (SMT medical, Wuerzburg, Germany), which assessed changes in arterial stiffness by measuring pulse wave velocity (PWV) as recommended by the American Heart Association in 2015 [25]. A standard 10 cm pressure cuff was placed on the upper right thigh as high as possible towards the crotch while a 7 cm pressure cuff was wrapped around the wrist of the same arm. The cuff was closed tight enough to assure a good coupling of the cuff to the femoral artery. The right common carotid artery pulse was palpated on the centre between the base of the neck and chin and a neck band with an attached neck pressure cuff was placed snugly around the neck with the cuff-bladder exactly over the palpated carotid artery pulse. Pressure lines were attached to the cuffs and the test conducted by recording the PWV (m/s) at the carotid and femoral arteries. The resulting PWV was defined as the ratio of pulse wave transit time and distance between these two vessels.

### 2.7. Sample Collection and Biochemical Analysis

Urine was collected from all participants in sterile tubes and was used to quantify the following biochemical parameters. Creatinine was quantified using the Roche Cobas 6000 analyser while albumin, asymmetric dimethylarginine (ADMA) and 8-Hydroxy-2′-deoxyguanosine (8-OHdG) were assayed using ELISA kits (Elabscience, Houston, Texas, USA) according to manufacturer’s protocol. Lipid peroxidation assay was performed based on the quantification of thiobarbituric acid reactive substances (TBARs) as described by Mallick and colleagues [26].

### 2.8. Statistical Analysis

Data were analysed using STATA MP version 14.1 (College Station, TX, USA). Results were presented as mean ± confidence interval (CI). An analysis of variance (ANOVA) test was used to compare the mean differences of study parameters based on location and sex. A Jonckheere–Terpstra trend test was used to determine if there was a statistically significant trend between ordinal independent variables and dependent continuous variables. Pearson correlation was used to evaluate the relationship of cardiovascular risk factors with PWV and ADMA. A *p*-values ≤ 0.05 was considered significant.

## 3. Results

### 3.1. General Characteristics of Study Participants

Three hundred and six (306) children were recruited for the study which included 152 children from rural area and 154 from urban area. Among the 152 children in the rural area, there were 83 girls and 69 boys, while among the 154 children in urban area, there were 88 girls and 66 boys. Although the data are presented for girls and boys, the statistical comparisons are between urban and rural. Age, height and weight were similar between urban and rural children. NC, MUAC, TC and CC were significantly (*p* < 0.05) higher in urban than rural children. The WC, BMI and AC were similar between urban and rural children. Rural children had significantly (*p* < 0.001) higher SBP and PWV than urban children, while DBP, MAP and HR were significantly (*p* < 0.001) different between urban and rural children. Albumin, ACR, TBARS and ADMA were significantly (*p* < 0.001) higher in rural children than in urban children, while creatinine and 8-OHdG were significantly (*p* < 0.001) higher in urban children than in rural children (Table 1).

### 3.2. Prevalence of Overweight, Obesity, Pre-Hypertension and Hypertension among Participating Children

Children were separated by location and sex to determine the prevalence of overweight, obesity, pre-hypertension and hypertension as shown in Table 2. The prevalence of obesity was 7.5% and was higher in children from urban settings (4.2%) compared to those from rural settings (3.3%). Overweight/obesity was more prevalent in girls than in boys. The prevalence of hypertension was 10.5% and was higher in rural children (5.9%) than in urban children (4.5%). The prevalence of pre-hypertension/hypertension in rural girls (14.7%) almost doubled that of rural boys (8.5%), while the prevalence of pre-hypertension/hypertension in urban settings was the same (9.5%) in both girls and boys.

### 3.3. Effect of Pulse Wave Velocity on Cardiovascular Risk Factors

Children were classified into four inter-quartile ranges of their PWV in order to determine the effect of PWV on cardiovascular risk factors. MAP and DBP increased with increasing PWV and the differences were significant (*p* < 0.05) for DBP. Jonckheere–Terpstra trend test showed a trend of MAP and DBP to increase with increasing PWV (*p* < 0.05). However, SBP and HR did not show any specific pattern with the increasing quartiles of PWV. Similarly, BMI, ADMA, 8-OHdG, TBARS, creatinine, albumin and ACR were not significantly (*p* > 0.05) different among the PWV quartiles (Table 3).

### 3.4. Effect of Asymmetric Dimethylarginine on Cardiovascular Risk Factors

There are currently no cut-off values for ADMA in children. In order to study the effect of ADMA on cardiovascular risk factors, children were classified into inter-quartile ranges of ADMA. As confirmed by the Jonckheere–Terpstra trend test, DBP, creatinine, albumin, ACR and 8-OHdG significantly (*p* < 0.001) increased with increasing quartiles of ADMA. SBP and HR insignificantly (*p* > 0.05) increased with increasing quartiles of ADMA. Moreover, BMI, MAP and TBARS did not show any significant (*p* > 0.05) specific pattern of distribution (Table 4).

### 3.5. Relationship between Endothelial Function Markers and Cardiovascular Risk Factors

PWV positively correlated (*p* < 0.05) with BMI, DBP and MAP while ADMA showed a positive correlation (*p* < 0.05) with creatinine and 8-OHdG. PWV and ADMA did not correlate with other cardiovascular risk factors, including SBP, HR, albumin, ACR and TBARS. Results are summarised in Table 5.

## 4. Discussion

There is evidence of an increased prevalence of cardiovascular risk factors, especially obesity and hypertension, in children [27,28]. Childhood obesity is of major concern in South Africa. A study conducted among children aged 7–10 years old in Port Elizabeth, South Africa, showed 20.9% and 9.8% prevalence of overweight and obesity, respectively [29]. In this present study, the prevalence of obesity was 7.5% and was higher in girls than boys. Possible reasons for the overall higher prevalence of overweight/obesity in girls could be due to biological and socio-cultural differences as has been previously reported [30,31]. In addition, the prevalence of overweight/obesity was higher in urban children (10.8%) than their rural counterparts (8.5%). This finding is in agreement with a previous study among South African children aged 0–18 years old by Monyeki and colleagues who reported a higher prevalence of obesity in urban (6.1%) than in rural (3.7%) children [32]. The differences in the prevalence of obesity between urban and rural children might be as a result of age difference as well as lifestyle differences between urban and rural children.

High blood pressure, a condition associated with obesity, is becoming more prevalent in children [33,34]. Our findings showed 10.5% prevalence of high blood pressure in children. The high blood pressure observed among school-going children in this study is not a new phenomenon in Africa though the prevalence of high blood pressure in the present study is higher compared to other studies. A prevalence of 9.8% for high blood pressure was shown in a study conducted in Gambia in children aged 5–9 years old with females presenting higher prevalence [34]. Another study conducted in urban and rural settings of Der es Salaam in Tanzania showed a 15.2% prevalence of elevated/high blood pressure [33]. This present study showed high prevalence of elevated/high blood pressure in children from rural (23.2%) areas as compared to their urban counterparts (19.0%). More so, blood pressure measures of children from rural areas were significantly (*p* < 0.05) higher than that of urban areas. Obesity and hypertension are known to co-exist, but the pattern of distribution of obesity and hypertension in this study was different between rural and urban children. Although this observation remains unclear and we may be tempted to rule out feeding habits as a possible reason for this observation, differences in the consumption of salt may likely be implicated. 

Vascular dysfunction, which is an early indicator of CVDs [35], has been shown to be associated with known cardiovascular risk factors including obesity, hypertension, oxidative stress, dyslipidaemia and microalbuminuria [16,35]. Though there is evidence of increased prevalence of cardiovascular risk factors in children, limited data are available on their relationship with vascular dysfunction in South Africans of African ancestry. Vascular function is strongly dependent on the endothelium, which is a vascular barrier that is important in the regulation of blood flow into micro- and macrovascular circulation [36]. Endothelial cells lining the arterial lumen are known to regulate vascular tone and also maintain vascular homeostasis by keeping a delicate balance between vasodilation and vasoconstriction [36]. The endothelial function of vasodilation and vasoconstriction is regulated by nitric oxide (NO). Alteration in the production of NO impairs the functioning of the endothelium leading to endothelial dysfunction [37], a condition which impairs vasodilation [38]. On the other hand, ADMA is a molecule that inhibits eNOS from synthesizing NO from L-arginine and, thus, increased ADMA leads to reduced production of NO [38]. ADMA has been shown to be associated with endothelial dysfunction in healthy individuals [39]. Arterial stiffness, which is the distensibility of muscular conduit arteries, is an early sign of endothelial dysfunction. PWV, which is used to assess arterial stiffness, has been shown to measure flow-mediated changes in vascular tone and, thus, is used as a surrogate method to assess endothelial function [40].

Impairment of vasodilation due to endothelial dysfunction results in the constriction of blood vessels (arterial stiffness) and eventually leads to hypertension. It has been reported that endothelial dysfunction is associated with a sustained increase in blood pressure [41]. Uncontrolled hypertension results in subclinical structural changes in the cardiovascular system measured as cardiac wall thickening, carotid intima-media thickness and arterial stiffness characterised by increased PWV [42]. In this study, blood pressure measures increased with increasing quartiles of PWV and ADMA. In addition, DBP positively associated with PWV. This finding concords with a study conducted by Kulsum-Mecci and colleagues [43] which showed that PWV was significantly higher in hypertensive children aged 4–18 years old. It has been reported that high DBP signifies a risk of CVD, as a pulse wave is reflected during diastole, causing the heart to work harder [44]. Additionally, a baseline survey which was conducted with 6–15-year-old children in 1987 in Hanzhong city who were followed up for 26 years showed that children with high blood pressure had a significantly higher incidence of hypertension and brachial-ankle PWV in their adulthood than the normotensive children [45]. These reports suggest that hypertension may result in vascular dysfunction in children.

Obesity is another factor that has been suggested to be linked with vascular dysfunction. In obesity, there is adipocyte hypertrophy which leads to excess production of pro-inflammatory markers such as chemokines, adipokines and cytokines [46]. These inflammatory mediators including interleukin 6 (IL-6) and tumour necrosis factor-a (TNF-α) secreted from adipocytes are responsible for decreasing the production and secretion of adiponectin [47], a molecule that increases the production of NO and promotes endothelial function [48]. Therefore, obesity may impair vascular function. Findings from this study showed that BMI was positively associated with PWV. Previous studies have shown obesity to induce vascular dysfunction [49].

Oxidative stress is another factor that may be related to vascular dysfunction. Oxidative stress is a result of excessive levels of ROS that overwhelms the antioxidant system. These excessive ROS, such as superoxide, can react with NO to form peroxynitrite (ONOO-) [50], thereby reducing the available NO level for proper endothelial function. Thus, high ROS generation may lead to different abnormalities including endothelial dysfunction [51,52]. Thiobarbituric acid reactive substances (TBARS), which result from cell membrane damage and 8-hydroxyl-2-deoxy guanosine (8-OHdG)—formed as a result of DNA damage—are common markers of oxidative stress [53]. Findings from this study showed 8-OHdG, a marker for oxidative stress to significantly increase with increasing quartiles of ADMA and also positively correlated with ADMA. More so, 8-OHdG was shown to predict endothelial dysfunction in children. Although not in children, previous studies have shown oxidative stress to be associated with endothelial dysfunction [54,55,56].

Microalbuminuria is known to be an independent risk factor of CVDs [57]. Microalbuminuria, which can reliably be defined by elevated ACR [58], has been shown to be associated with vascular dysfunction [14]. Thus, there is a possibility that vascular dysfunction is related to microalbuminuria in children. Creatinine, albumin and ACR significantly increased with increasing ADMA concentration, and ADMA positively associated with creatinine. These findings suggest a possible association of microalbuminuria with vascular dysfunction in children. Urine ACR has been shown to be an early marker for vascular dysfunction in adolescents independent of glycaemia [58]. In addition, microalbuminuria has been shown to positively associate with endothelial dysfunction in HIV-infected patients regardless of known confounders [57]. This study has identified possible cardiovascular risk factors of vascular dysfunction in children and has provided initial information for further studies. However, these findings may be limiting since it was a cross-sectional study and, therefore, causal relationship between these risk factors and vascular dysfunction may not fully be established. Also, the biomarkers for cardiovascular risk were assayed in urine and therefore, may not strictly reflect measured outcomes in blood.

## 5. Conclusions

Vascular dysfunction was associated with obesity, high blood pressure, oxidative stress and microalbuminuria in South African children of African ancestry. Overweight/obesity was more prevalent in urban children while elevated/high blood pressure was more prevalent in rural children. Our findings suggest that South African children of African ancestry may be at risk of developing CVDs. Thus, there is need for intervention strategies specific for children of African ancestry to be instituted to prevent future development of cardiovascular complications.

## Figures and Tables

**Table 1:** General characteristics of participating children by sex and location.

	Rural (95% CI)		Urban (95% CI)		*p*-Value
	Girls	Boys	Girls	Boys	
*N*	83	69	88	66	
Age (years)	7.9 (7.7–8.2)	7.9 (7.6–8.2)	8.3 (8.1–6.7)	8.1 (7.7–8.5)	0.320
HT (cm)	124.8 (122.9–126.7)	126.9 (124.4–129.3)	129.8 (125.3–134.3)	127.9 (125.0–130.1)	0.198
WT (kg)	25.4 (24.4–26.4)	27.5 (25.1–29.8)	28.6 (26.6–30.6)	28.1 (26.0–30.2)	0.137
BMI (m^2^/kg)	16.4 (15.8–16.9)	16.8 (15.8–17.9)	17.2 (16.4–18.0)	17.1 (16.0–18.2)	0.276
NC (cm)	24.9 (24.5–25.3)	26.2 (25.6–26.7)	26.0 (25.4–26.5)	26.6 (25.9–23.4)	<0.001
MUAC (cm)	19.3 (18.8–19.8)	19.4 (18.5–20.2)	20.5 (19.7–21.3)	20.3 (19.4–21.2)	<0.01
WC (cm)	58.8 (57.4–60.3)	59.6 (56.9–61.2)	58.5 (56.5–60.5)	57.9 (55.3–60.5)	0.184
TC (cm)	34.2 (33.2–35.3)	36.1 (34.4–37.7)	39.6 (38.1–41.1)	35.9 (33.5–38.3)	<0.001
CC (cm)	24.4 (23.7–25.1)	25.5 (24.5–26.5)	26.9 (25.9–27.8)	26.6 (25.4–27.7)	0.01
AC (cm)	18.3 (17.9–18.7)	19.1 (18.4–19.8)	18.6 (17.9–19.3)	18.9 (18.1–19.9)	0.514
SBP (mmHg)	108.8 (106.1–111.6)	107.9 (104.0–111.9)	108.4 (104.9–111.8)	107.7 (104.7–110.0)	<0.001
DBP (mmHg)	71.1 (69.1–73.1)	68.4 (65.9–71.0)	69.3 (66.9–71.7)	69.5 (66.2–72.9)	<0.001
HR (bpm)	89.8 (86.3–93.3)	89.4 (86.3–92.5)	93.2 (90.4–96.0)	87.6 (84.4–90.8)	<0.001
MAP (mmHg)	83.7 (81.6–85.7)	82.2 (79.9–84.5)	82.3 (79.8–84.9)	82.5 (79.5–85.5)	<0.001
PWV (m/s)	4.8 (4.6–5.0)	8.1 (1.5–14.8)	4.7 (4.4–5.0)	8.0 (1.7–14.3)	<0.001
Creatinine (mmo/L)	7.2 (6.2–8.1)	8.7 (6.5–10.8)	10.8 (9.0–12.6)	8.5 (6.5–10.4)	<0.001
ADMA (ng/mL)	72.1 (68.8–75.4)	76.4 (73.8–79.6)	75.1 (72.6–77.7)	73.2 (66.3–80.0)	<0.001
Albumin (mg/L)	47.1 (−7.5–101.7)	38.7 (−9.8–87.1)	41.8 (−9.1–92.7)	5.0 (2.7–7.4)	<0.001
ACR (mg/mmol)	6.2 (−0.1–12.3)	3.4 (−0.9–7.7)	4.2 (−1.5–9.9)	0.6 (0.4–0.8)	<0.001
TBARS (µM)	0.08 (0.07–0.08)	0.09 (0.05–0.12)	0.08 (0.06–0.09)	0.07 (0.05–0.08)	<0.001
8-OHdG (ng/mL)	61.6 (57.4–65.9)	66.7 (58.3–74.9)	64.9 (60.2–69.6)	65.5 (59.6–71.5)	<0.001

Results are expressed as mean (min CI–max CI); CI: confidence interval; N: number of children; HT: height; WT: weight; BMI: body mass index; NC: neck circumference; MUAC: mid upper arm circumference; WC: waist circumference; TC: thigh circumference; CC: calf circumference; AC: ankle circumference; SBP: systolic blood pressure; DBP: diastolic blood pressure; HR: heart rate; MAP: mean arterial pressure; PWV: pulse wave velocity; ADMA: asymmetric dimethylarginine; ACR: albumin to creatinine ratio; TBARS: thiobarbituric acid reactive substances; 8-OHdG: 8-hydroxyl-deoxy-guanosine.

**Table 2:** Prevalence of overweight, obesity, pre-hypertension and hypertension.

	Cohort (%)		Rural (%)		Urban (%)	
	Rural	Urban	Girls	Boys	Girls	Boys
Overweight	16 (5.2)	20 (6.6)	10 (3.2)	6 (1.9)	12 (3.9)	8 (2.6)
Obesity	10 (3.3)	13 (4.2)	7 (2.3)	3 (1.0)	8 (2.6)	5 (1.6)
Overweight/Obesity	26 (8.5)	33 (10.8)	17 (5.55)	9 (2.9)	20 (6.5)	13 (4.2)
EBP	53 (17.3)	44 (14.3)	33 (10.8)	20 (6.5)	20 (6.6)	24 (7.9)
HBP	21 (6.9)	11 (3.6)	12 (3.9)	6 (2.0)	9 (2.9)	5 (1.6)
EBP/HBP	71 (23.2)	58 (19.0)	45 (14.7)	26 (8.5)	29 (9.5)	29 (9.5)

EBP: elevated blood pressure; HBP: high blood pressure; percentage (%) indicates prevalence.

**Table 3:** Effect of pulse wave velocity on cardiovascular risk factors.

PWV Quartiles	1st Quartile (95% CI)	2nd Quartile (95% CI)	3rd Quartile (95% CI)	4th Quartile (95% CI)	*p*-Value
*N*	76	77	77	76	
BMI (m^2^/Kg)	16.3 (15.7–16.9)	16.2 (15.8–16.7)	16.3 (15.6–16.9)	16.9 (16.0–17.8)	0.495
SBP (mmHg)	110.8 (102.0–119.6)	107.9 (105.5–110.4)	107.8 (105.3–110.2)	109.5 (107.2–111.8)	0.822
DBP (mmHg)	68.1 (66.1–69.9)	67.0 (64.8–69.3)	68.4 (66.6–70.2)	72.4 (70.4–74.4)	0.001
HR (bpm)	89.1 (86.0–92.2)	89.6 (86.6–92.7)	91.7 (87.9–95.5)	90.3 (87.4–93.1)	0.716
MAP (mmHg)	81.9 (78.4–85.5)	81.7 (78.2–85.2)	80.2 (77.2–83.3)	83.4 (81.6–85.2)	0.532
ADMA (ng/mL)	67.8 (62.9–72.7)	71.7 (67.8–75.6)	72.4 (67.0–77.7)	70.2 (66.1–74.4)	0.494
8-OHdG (ng/mL)	64.4 (59.8–68.9)	74.1 (50.1–98.1)	62.8 (57.8–67.8)	72.4 (57.4–87.5)	0.621
TBARS (µM)	0.07 (0.06–0.08)	0.07 (0.07–0.08)	0.07 (0.06–0.08)	0.08 (0.06–0.09)	0.862
Creatinine (mmol/L)	7.7 (6.6–8.7)	7.6 (6.5–8.7)	7.8 (6.7–8.9)	7.4 (6.3–8.5)	0.957
Albumin (mg/L)	16.9 (3.6–30.3)	39.4 (4.1–74.7)	32.3 (−12.4–77.0)	27.8 (5.6–49.9)	0.734
ACR (mg/mmol)	2.9 (0.5–5.3)	5.4 (0.5–10.4)	4.3 (−0.2–8.8)	2.9 (0.8–5.1)	0.700

Results are expressed as mean (min CI–max CI); CI: confidence interval; *N*: number of children; BMI: body mass index; SBP: systolic blood pressure; DBP: diastolic blood pressure; HR: heart rate; MAP: mean arterial pressure; PWV: pulse wave velocity; ADMA: asymmetric dimethyl arginine; ACR: albumin to creatinine ratio; TBARS: thiobarbituric acid reactive substances; 8-OHdG: 8-hydroxyl-deoxy-guanosine. PWV quartiles (m/s): 1st quartile: <3.38; 2nd quartile: 4.1 and 5.20; 3rd quartile: 5.3 and 5.7; 4th quartile: >6.10.

**Table 4:** Effect of asymmetric dimethylarginine on risk factors of cardiovascular diseases.

ADMA Quartiles	1st Quartile (95%CI)	2nd Quartile (95%CI)	3rd Quartile (95%CI)	4th Quartile (95%CI)	*p*-Value
*N*	47	48	47	48	
BMI (m^2^/Kg)	17.5 (16.2–18.8)	16.4 (15.9–16.9)	17.1 (15.9–18.3)	16.6 (16.1–17.2)	0.503
SBP (mmHg)	100.5 (100.3–100.8)	107.9 (104.8–111.0)	108.0 (104.7–111.2)	108.3 (105.2–111.4)	0.98
DBP (mmHg)	68.1 (65.9–70.2)	68.7 (66.4–70.9)	69.8 (67.5–72.1)	70.2 (68.5–71.9)	0.036
HR (bpm)	89.8 (85.3–94.4)	90.5 (86.2–94.9)	90.8 (86.0–95.5)	91.3 (87.5–93.6)	0.97
MAP (mmHg)	85.9 (82.8–88.9)	82.8 (79.7–85.9)	82.2 (70.2–84.8)	82.0 (80.2–83.9)	0.664
8-OHdG (ng/mL)	64.6 (36.4–92.9)	65.2 (55.3–75.0)	66.7 (63.3–70.1)	77.9 (65.6–90.1)	<0.001
TBARS (µM)	0.07 (0.06–0.08)	0.08 (0.06–0.11)	0.07 (0.06–0.07)	0.07 (0.06–0.08)	0.820
Creatinine (mmol/L)	5.6 (4.3–6.9)	9.1 (7.1–11.1)	9.7 (8.2–11.1)	7.3 (6.5–8.2)	<0.001
Albumin (mg/L)	6.0 (2.7–5.3)	22.0 (8.9–35.1)	36.9 (−7.1–80.9)	35.4 (−0.7–71.5)	0.016
ACR (mg/mmol)	1.2 (0.5–1.9)	2.1 (1.3–2.9)	3.8 (−1.2–8.7)	4.6 (0.6–8.6)	0.022

Results are expressed as mean (min CI–max CI); CI: confidence interval; N: number of children; BMI: body mass index; SBP: systolic blood pressure; DBP: diastolic blood pressure; HR: heart rate; MAP: mean arterial pressure; ADMA: asymmetric dimethylarginine; ACR: albumin to creatinine ratio; TBARS: thiobarbituric acid reactive substances; 8-OHdG: 8-hydroxyl-deoxy-guanosine. ADMA quartiles (ng/mL): 1st quartile: <68.00; 2nd quartile: 68.4 and 75.1; 3rd quartile: 75.2 and 79.58; 4th quartile: >79.06.

**Table 5:** Correlation between endothelial function markers and cardiovascular risk factors.

	ADMA		PWV	
	*r*	*p*-Value	*r*	*p*-Value
BMI (m^2^/Kg)	−0.04 (−0.21–0.11)	0.674	0.28 (−0.37–0.60)	0.002
SBP (mmHg)	0.08 (−0.1–0.24)	0.461	0.12 (−0.04–0.28)	0.184
DBP (mmHg)	−0.06 (−0.19–0.07)	0.482	0.27 (0.07–0.45)	0.002
HR (bpm)	0.01 (−0.19–0.19)	0.991	−0.02 (−0.25–0.19)	0.809
MAP (mmHg)	−0.12 (−0.26–0.02)	0.174	0.23 (0.04–0.41)	0.011
Creatinine (mmol/L)	0.27 (0.11–0.39)	0.003	−0.02 (−0.21–0.16)	0.797
Albumin (mg/L)	0.12 (0.03–0.19)	0.195	−0.01 (−0.09–0.09)	0.985
ACR (mg/mmol)	0.12 (−0.01–0.20)	0.207	−0.03 (−0.11–0.06)	0.711
8-OHdG (ng/mL)	0.51 (0.38–0.64)	0.001	0.13 (−0.02–0.25)	0.164
TBARS (µM)	−0.03 (−0.19–0.09)	0.742	0.11 (−0.10–0.26)	0.225

*r*: correlation coefficient; BMI: body mass index; SBP: systolic blood pressure; DBP: diastolic blood pressure; HR: heart rate; MAP: mean arterial pressure; BMI: body mass index; PWV: pulse wave velocity; ADMA: asymmetric dimethylarginine; ACR: albumin to creatinine ratio; TBARS: thiobarbituric acid reactive substance; 8-OHdG: 8-hydroxyl-deoxy-guanosine.

## Data Availability

We do not wish to share the data included in this manuscript as the participants data are kept confidential in accordance with the South African National Data Protection guidelines of reporting.
